# BioEMMA: Automated Generation of Model-Specific Escher-Compatible Maps from KEGG Pathways

**DOI:** 10.3390/biology15171493

**Published:** 2026-09-02

**Authors:** Vladislav A. Kachnov, Maryam A. Esembaeva, Ekaterina V. Melikhova, Mikhail A. Kulyashov

**Affiliations:** Department of Computational Biology, Scientific Center of Genetics and Life Sciences, Sirius University of Science and Technology, 354340 Sirius, Russia; kachnov.va@talantiuspeh.ru (V.A.K.); esembaeva.ma@talantiuspeh.ru (M.A.E.); melihova.ev@talantiuspeh.ru (E.V.M.)

**Keywords:** genome-scale metabolic models, metabolic pathway visualization, metabolic model reconstruction, flux balance analysis, KEGG pathways, Escher maps, model comparison

## Abstract

Genome-scale metabolic models are widely used to investigate cellular metabolism, but their interpretation and comparison are limited by the lack of reproducible pathway-level visualizations with a common spatial organization. This study presents BioEMMA, a Python-based tool for the automated generation of model-specific metabolic pathway maps in the Escher JSON format using coordinate information from curated KEGG pathway maps. BioEMMA parses KGML files, map reaction and metabolite identifiers to model database namespaces, filters pathway elements according to an input SBML model, adds non-primary metabolites, reconstructs Escher-compatible layouts, and supports flux visualization. The tool was integrated into a reproducible BioUML workflow for metabolic model reconstruction. BioEMMA was evaluated using the e_coli_core model and the KEGG glycolysis/gluconeogenesis pathway while generating a model-specific map with overlaid FBA fluxes. It was then applied to compare *E. coli* reconstructions generated by gapseq, ModelSEEDpy, and Reconstructor across three central carbon metabolism pathways. To broaden the evaluation, BioEMMA was applied using 87 prokaryotic BiGG models and three eukaryotic models. The analysis revealed pathway-specific differences in reaction coverage, shared and model-specific reactions, and predicted flux activity. BioEMMA therefore provides a reproducible framework for pathway-level visualization and comparison of genome-scale metabolic reconstructions within a common spatial coordinate system.

## Simple Summary

Computer models of cell metabolism can contain thousands of reactions and are therefore difficult to inspect directly. BioEMMA was developed to convert such models into clear pathway maps without requiring researchers to draw each map manually. A user selects a biological pathway and provides a metabolic model, after which the main conversion and matching steps are performed automatically. BioEMMA can be run independently or included in a standardized and reproducible computational workflow that links genome processing, model reconstruction, quality assessment, and visualization. The resulting maps can be opened in Escher, an interactive pathway editor, where researchers can examine predicted reaction activity, correct labels or local connections, and refine the map for further analysis. Tests with bacterial models showed that these maps help reveal missing reactions, model-specific pathway regions, and differences in predicted flux distribution. BioEMMA therefore provides researchers with a practical starting point for inspecting, comparing, and improving metabolic models while reducing the time required for manual map preparation.

## 1. Introduction

Genome-scale metabolic (GSM) models are a powerful framework in systems biology, which gives an opportunity for linking genome annotation to biochemical reaction networks and enabling constraint-based analyses, such as flux balance analysis (FBA), to investigate the growth, metabolic phenotypes, and biotechnologically relevant properties of microorganisms [1,2]. The rapid accumulation of sequencing data, particularly for non-reference bacterial isolates, has increased the need for reproducible workflows that convert genomic data into simulation-ready SBML/GSM models. Although several automated reconstruction tools have been developed, including ModelSEED (and its Python implementation, ModelSEEDpy), Kbase, CarveMe, gapseq, and Reconstructor, models reconstructed from the same genome may differ substantially because of differences in reaction databases, annotation rules, gene–protein–reaction assignment, and gap-filling strategies [3,4,5,6,7]. Therefore, practical use of GSMs requires not only automated reconstruction but also standardized evaluation of model quality and consistency, as implemented in the MEMOTE framework [8].

However, numerical quality metrics alone do not always provide a biological interpretation of differences between alternative reconstructions. Interpreting reaction content, flux distributions, and model-specific differences requires visualization in the context of metabolic pathways. Escher is one of the most widely used tools for building and editing pathway maps and overlaying reaction, metabolite, and gene-associated data [9], while Escher–FBA further connects such maps with interactive FBA simulations [10]. Nevertheless, Escher maps often require manual construction or adaptation to a specific model, which limits scalability when many reconstructions or alternative models need to be compared.

Recent GSM visualization tools, including SAMMI, Fluxer, MetExplore, CAVE, and NAViFluX, reflect a broader trend toward integrated environments that combine pathway visualization, flux analysis, model refinement, and omics data overlay [11,12,13,14,15]. Many automated approaches, however, rely on de novo or graph-based layouts. Although useful for exploring network structure, such layouts do not necessarily preserve the familiar biological topology of curated pathway maps. This becomes a limitation when comparing models reconstructed using different tools, because node coordinates and overall pathway organization may vary between visualizations.

Here, we introduce BioEMMA (BioUML Escher Metabolic Maps Assistant), a tool for generating model-specific, Escher-compatible metabolic maps from curated KEGG pathway definitions. This study was designed to determine whether the reaction content of a genome-scale metabolic model can be represented within a stable reference pathway layout and whether this representation facilitates the comparison of independently reconstructed models. BioEMMA was first evaluated against a reference model for which a manually curated Escher map was available and was then used to compare alternative reconstructions generated from the same genomic input. To extend the evaluation beyond a single organism and reconstruction setting, we analyzed four KEGG pathways across 87 prokaryotic BiGG models, including 57 *Escherichia coli* models, and 3 eukaryotic models, assessing reaction retention and between-model map similarity. Finally, a reproducible workflow for genome processing, metabolic model reconstruction, quality assessment, and pathway visualization was developed within the BioUML environment, with BioEMMA integrated as its final visualization component.

## 2. Materials and Methods

### 2.1. BioEMMA: Automatic Generation of Escher-Compatible Metabolic Maps

**Figure 1:**
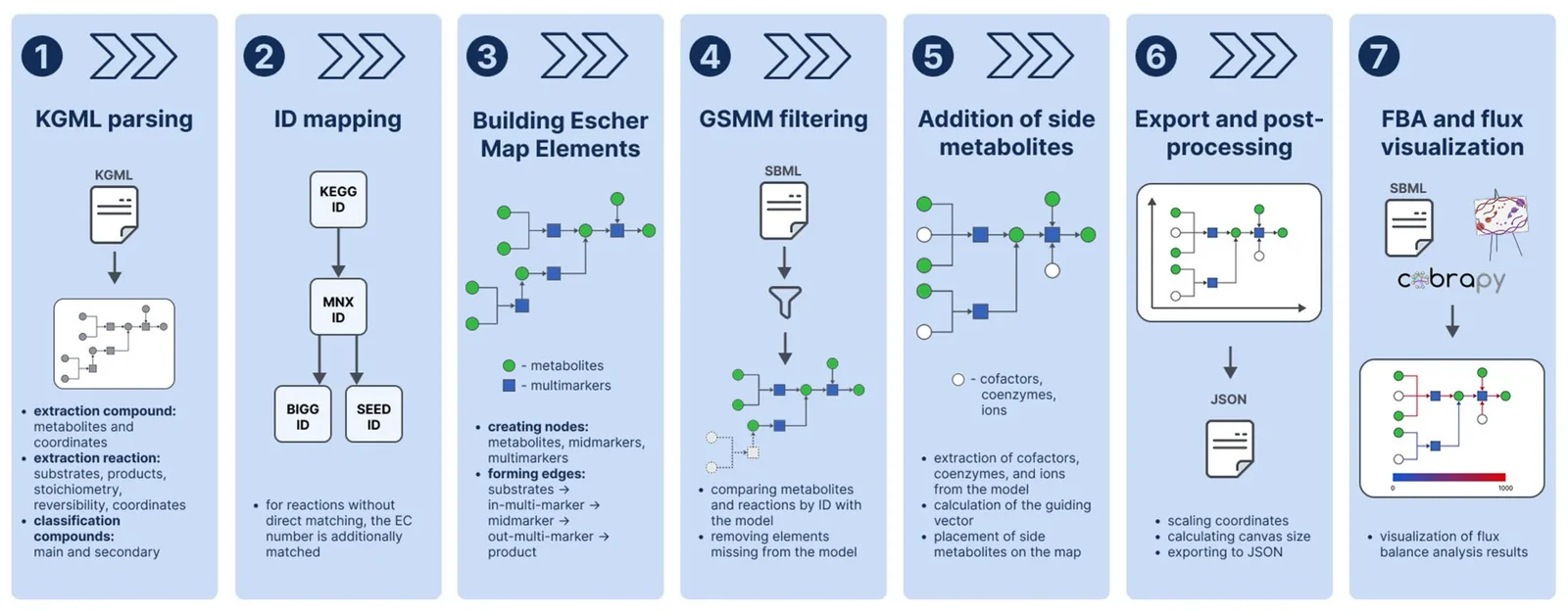
Schematic representation of BioEMMA workflow.

For the automated visualization of the reconstructed model, the BioEMMA tool (BioUML Escher Metabolic Maps Assistant) was developed. BioEMMA converts KEGG pathway metabolic maps into a JSON representation compatible with the Escher Map format [9]. The tool was implemented in Python using the numpy (v2.4.4) and BeautifulSoup (v4.14.3) libraries. The source code is available in the GitHub repository (https://github.com/KAVladD/BioEMMA, accessed on 15 August 2026), the installation package is published on PyPI (https://pypi.org/project/bioemma/, accessed on 15 August 2026), and the Docker image is hosted on Docker Hub (https://hub.docker.com/r/developmentontheedge/bioemma, accessed on 15 August 2026) to enable the use of BioEMMA as part of reproducible computational workflows. The BioEMMA workflow includes reading a KEGG KGML map, mapping reaction and metabolite identifiers to the namespaces of model databases, filtering map elements according to the composition of the target metabolic model, converting KGML-derived coordinates into Escher-compatible map elements, and exporting the result for subsequent analysis or visualization of flux data. Figure 1 provides an overview of the BioEMMA workflow. To clarify how the input pathway definition is transformed into a model-specific Escher-compatible map, each processing stage is described below in the order in which it is executed.

1. **Parsing of the KEGG metabolic map.** A metabolic pathway file in KGML format is provided as input. The XML parser extracts two types of entries: compound entries (metabolites) and reaction entries (enzymatic reactions). For each metabolite, the coordinates from the KGML file corresponding to its position on the original KEGG map are retained. For each reaction, the lists of substrates and products with their stoichiometric coefficients, the reversibility attribute, and the coordinates of the reaction position on the map are extracted. Metabolites explicitly present in the compound section are classified as primary, whereas the remaining reaction participants, including cofactors and coenzymes, are treated as non-primary metabolites.
2. **Cross-reference mapping of identifiers.** Metabolite and reaction identifiers from the KEGG namespace [16] are translated into identifiers of the target database, BiGG or ModelSEED [3,17] using precompiled cross-reference tables obtained from the MetaNetX (v 4.5) database [18]. Compound and reaction identifiers were processed separately: each KEGG compound identifier was linked to the corresponding MetaNetX chemical record (MNXM), whereas each KEGG reaction identifier was linked to a MetaNetX reaction record (MNXR). Cross-references from the resulting MetaNetX record to the selected target database were then retrieved. This procedure represents a deterministic lookup of existing MNXref correspondences and does not involve the prediction of new mappings. If MNXref contained no cross-reference from a KEGG record to the selected target database, BioEMMA did not assign a BiGG or ModelSEED identifier to that record. Consequently, a KEGG reaction without such a correspondence could not be matched to a reaction in the source model through the direct-mapping procedure and was therefore not retained as a model-associated reaction on the generated map.
  BioEMMA also provides an optional EC-based fallback for unresolved reaction identifiers. When enabled, this procedure considers candidate reactions from the target database that share at least one EC number with the KEGG reaction. Candidates are ranked according to the overlap between their sets of mapped reaction participants, calculated as the number of shared participants divided by the size of the smaller participant set. The highest-scoring candidate is retained if its overlap score is at least 0.5. Equally scoring candidates are reported as ambiguous matches. This option is disabled by default and was not used in the analyses reported in this study. Because the fallback infers correspondences from partial EC number and participant agreement, it may introduce false positive mappings. We therefore recommend using this option with caution and manually reviewing all fallback-derived matches before including them in downstream analyses.
3. **Construction of Escher map elements.** The parsed data are used to construct a map graph in the Escher Map JSON format. For each metabolite, a metabolite-type node is generated using the coordinates obtained from KGML. For each reaction, a midmarker-type node, representing the central reaction node, and a pair of multimarker-type nodes, representing the input and output connectors, are created. The positions of the multimarkers are calculated using a three-level heuristic. If all reaction metabolites lie on the same axis, either horizontal or vertical, the multimarker is placed on that axis with an offset from the reaction position toward the corresponding metabolite group. If at least one metabolite is aligned with the reaction along one coordinate, the multimarker is placed on the corresponding axis. In all other cases, the position is calculated by interpolation between the reaction position and the center of mass of the metabolite group. As a result, all nodes are assigned unique numerical indices within a single global namespace: metabolites are indexed first, followed by reaction midmarkers and then multimarkers. Edges (segments) are created between consecutive nodes according to the following sequence: substrate → input multimarker → midmarker → output multimarker → product.
4. **Filtering according to the genome-scale metabolic model.** An organism-specific GSM model in SBML format is provided as input. Each map reaction and metabolite are matched to the model using multiple identifiers: KEGG IDs, BiGG IDs, and ModelSEED IDs are compared with the annotations of the corresponding model objects. Map elements not found in the model are excluded from the final structure.
5. **Mapping of non-primary metabolites.** For each reaction retained after filtering, metabolites absent from the set of primary map metabolites, including cofactors, ions, and coenzymes, are extracted from the model. For these metabolites, metabolite-type nodes are generated with the attribute node_is_primary = False. The positions of non-primary metabolites are calculated relative to the corresponding multimarker. A direction vector is determined from the reaction position to the center of mass of the primary metabolites on the same side of the reaction, either substrates or products, after which the non-primary metabolites are placed along a line perpendicular to this vector, with a lateral offset proportional to the metabolite index.
6. **Export and postprocessing.** Before export, the coordinates of all nodes and labels are multiplied by the scaling_factor. The canvas dimensions are calculated from the maximum node coordinates with additional margins. All elements are centered relative to the canvas. The resulting structure is saved as a two-element JSON array comprising the map metadata and the model, which contains the nodes, reactions, text_labels, and canvas dictionaries. The map can be loaded into the Escher web application or used programmatically through the escher-py library for further modification and/or analysis of flux distributions after model optimization. When several input maps are processed, each map is first constructed and saved separately, after which the internal identifiers of nodes, reactions, and segments are converted to sequential numbering. The maps are then placed into the cells of a square grid of size ceil(sqrt(N)), where N is the number of maps. The cell size is determined from the maximum dimensions among all maps. The coordinates of nodes and reaction labels for each map are shifted by the corresponding offset to prevent overlap. The maps are then merged sequentially: new unique identifiers are assigned to all elements, and segment references to their initial and terminal nodes are updated according to the new numbering. After merging, the canvas is recalculated using the minimum and maximum coordinates of the resulting map with an additional 5% margin. Identical reactions or metabolites are not collapsed and are retained as separate elements belonging to their respective source maps.
7. **Visualization of flux balance analysis results.** In addition to metabolic map construction, BioEMMA supports model optimization using flux balance analysis (FBA) and visualization of the resulting fluxes directly on the generated map. For a specified GSM model in SBML format, optimization is performed using COBRApy (v0.31.1) [19], after which the calculated flux vector is passed to escher-py (v1.8.1). Fluxes are displayed on the map using a color scale and scaled edge thickness, and the resulting visualization is exported as an interactive HTML document. Thus, the entire workflow, from parsing the KEGG map to visualizing the results of flux analysis, is performed programmatically without manual interaction with the Escher web interface.

### 2.2. Integration of BioEMMA into BioUML Workflow Environment

**Figure 2:**
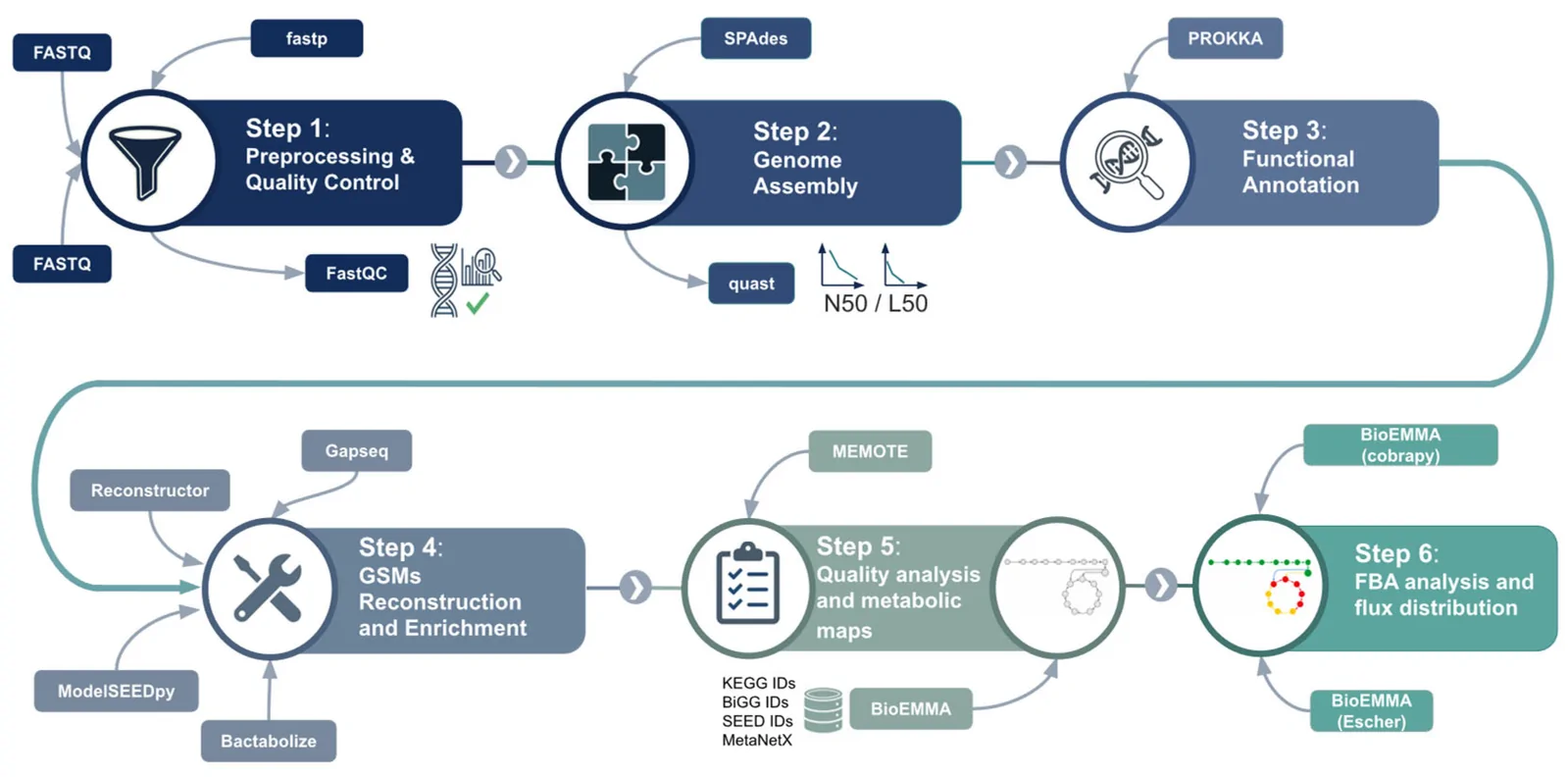
Schematic representation of implemented pipeline.

The BioEMMA algorithm described above was developed as a specialized module for the automated generation of model-specific metabolic pathway maps. In the present study, an automated pipeline for metabolic model reconstruction and analysis was also developed (Figure 2), in which BioEMMA performs the final stage after obtaining the model in SBML format. This architecture provides a continuous transition from model reconstruction to the generation of Escher-compatible maps and visualization of flux balance analysis results. All pipeline components, including BioEMMA, are packaged as Docker images and invoked through a unified WDL interface without manual interaction with the Escher web application.

The pipeline is implemented on the BioUML web platform [20], which provides integration with the Cromwell v83 workflow orchestration system. The platform supports parsing workflow descriptions written in WDL v1 (Workflow Description Language) and automatically generates a graphical interface based on these descriptions, allowing pipeline input parameters to be specified without directly editing WDL files. Each pipeline tool is executed as a separate WDL workflow within a Docker container. Some containers are used directly from the public Docker Hub registry: images from the StaPH-B project (https://hub.docker.com/u/staphb, accessed on 15 August 2026) are used for the preprocessing, genome assembly, and annotation stages, whereas the public cdiener/gapseq image (https://hub.docker.com/r/cdiener/gapseq, accessed on 15 August 2026) is used for gapseq. Containers for tools adapted or assembled specifically for this pipeline, including Bactabolize, Reconstructor, ModelSEEDpy, MEMOTE, and BioEMMA, are published on Docker Hub under the developmentontheedge namespace (https://hub.docker.com/u/developmentontheedge, accessed on 15 August 2026). In total, the pipeline includes eleven tools listed in Table 1. A detailed description of each tool, the corresponding WDL files, execution instructions, and a user guide for operating the pipeline within the BioUML platform are provided in the documentation: https://biouml-flux-modeling.readthedocs.io/en/latest/index.html, accessed on 15 August 2026.

The pipeline implements three usage scenarios that differ in the type of input data and the execution mode of the reconstruction tools. The WDL files for local execution of the implemented scenarios are available at https://github.com/mishakula-group/BioEMMA_article/tree/main/wdls, accessed on 15 August 2026.

Scenario I covers the complete workflow for processing genomic data starting from raw reads. The pipeline sequentially performs read preprocessing (fastp), quality control (FastQC), genome assembly (SPAdes), assembly quality assessment (QUAST), and annotation (Prokka). Based on the annotated genome, one of four reconstruction tools is launched according to the user’s selection: Bactabolize, ModelSEEDpy, Reconstructor, or gapseq. The reconstructed model is subjected to quality assessment using MEMOTE, followed by term enrichment and final visualization using BioEMMA.

Scenario II is intended for cases in which processed genomic data are already available. The pipeline accepts a completed genome assembly or an annotation file as input and starts directly from the reconstruction stage, bypassing the read-processing and assembly steps. Selection of the reconstruction tool, subsequent enrichment, quality assessment, and visualization are performed in the same manner as in Scenario I.

Scenario III implements multivariant parallel reconstruction: ModelSEEDpy, Reconstructor, and gapseq are run simultaneously on the same input data, either raw reads or a completed annotation. Bactabolize is not used in the parallel reconstruction mode because it requires a pre-existing pangenome model and, given the limited number of such models according to the tool documentation, it was decided not to include Bactabolize in this type of scenario. The pipeline branches into three independent workflows, each of which includes model reconstruction, quality assessment using MEMOTE, term enrichment, and map generation using BioEMMA. After all branches have been completed, an integrated comparative MEMOTE report is also generated, allowing differences between models obtained using different reconstruction tools to be evaluated according to a unified set of quality criteria.

### 2.3. Test Cases

Three complementary test cases were used to evaluate BioEMMA. The first assessed map generation using a reference model with an available curated Escher map, whereas the second examined its application to the pathway-level comparison of independently reconstructed genome-scale metabolic models, and the third evaluated BioEMMA across diverse prokaryotic and eukaryotic models.

#### 2.3.1. Test Case 1: Validation of BioEMMA on the Reference GSM Model

The *Escherichia coli* K-12 substr. MG1655 central metabolism model (e_coli_core), comprising 95 reactions and 72 metabolites, was used as the reference [17]. This model was selected because a manually curated reference map in the Escher format is available for it, enabling an assessment of the quality of automated map generation. The KEGG glycolysis/gluconeogenesis map (map00010) was selected for visualization and further comparison. BioEMMA was run using the map00010 KGML file, the SBML representation of the e_coli_core model, and tables mapping KEGG identifiers to the namespaces of model databases, followed by filtering of map elements according to the reactions and metabolites present in the target model. To overlay fluxes, the model was optimized using FBA under aerobic growth conditions on glucose as a single carbon source. The medium composition and exchange reaction constraints were specified in the flux calculation script, and optimization was performed using COBRApy v0.31.1. The resulting BioEMMA map was compared with the manually prepared e_coli_core Escher map, as well as with visualizations of the same model and the same set of FBA fluxes generated using NAViFLuX (GitHub version, commit 347790c, accessed 22 July 2026), SAMMI v0.1.7, Fluxer v2.10, CAVE (https://cave.biodesign.ac.cn/, accessed 22 July 2026), Grohar (https://github.com/mmoskon/Grohar, commit 4f4d287, accessed 22 July 2026) and MetExplore V2 v2.41.1 [11,12,13,14,15]. CytoSEED [27] was not included in the direct visualization comparison because it does not support models using BiGG identifiers. ModelExplorer [28] was also excluded because it accepts only SBML Level 2 models and does not support SBML Level 3 with the FBC package, which is currently used for constraint-based metabolic models. Therefore, both tools were assessed only on the basis of the capabilities reported in their respective publications. The comparison between tools was based on the following criteria: “Standard model format input” indicates support for common metabolic model formats such as SBML or JSON. “Pathway/subsystem-level visualization” denotes the ability to display biologically defined pathways or subnetworks. “Flux data overlay” indicates whether flux values or flux-derived states can be shown on the map. “Web-browser map interface” refers to interactive visualization in a browser-based HTML/JavaScript environment, excluding desktop-only tools. “Static map export” denotes export to figure-ready image formats such as SVG, PNG, JPEG, TIFF, or PDF. “Offline/local execution” indicates whether the core workflow can run locally without relying on a hosted service. “Programmatic access” denotes a documented non-GUI route such as an API, scripting interface, package, CLI, or plugin. “Automatic map generation” indicates automated construction of a map or graph from model, pathway, reaction, or flux input. “Native Escher-compatible JSON output” indicates direct export of maps in the Escher JSON schema. Full support, limited support, and no support were assigned based on whether each feature was directly available, available with important restrictions, or not supported/documented.

The BioEMMA execution parameters, FBA calculation scripts, execution parameters of the comparison tools, and the resulting maps and intermediate outputs are available in the project repository: https://github.com/mishakula-group/BioEMMA_article, accessed on 15 August 2026.

#### 2.3.2. Test Case 2: Use of BioEMMA in GSM Model’s Reconstruction Workflow

The second test case was designed to assess the applicability of BioEMMA to the comparison of alternative genome-scale reconstructions generated by different tools from the same input data and availability to use it as a part of reproducible workflow. Genome sequencing data for *Escherichia coli* str. K-12 *substr*. MG1655 obtained from the SRA database were used for model reconstruction within the pipeline: SRR13921546; project: PRJNA706563. Data processing was performed using Scenario III described above. We have chosen to demonstrate only scenario 3 because it includes both scenario 1 and scenario 2 and does not require their additional verification. As a result, after genome assembly and annotation, gapseq, ModelSEEDpy, and Reconstructor were run in parallel, and BioEMMA maps were generated for each resulting model. The “M9_vit_aa” medium from the set of media provided in the gapseq repository was used for gap filling (https://github.com/jotech/gapseq/tree/master/dat/media, accessed on 15 August 2026). The reconstruction tool parameters, WDL files, input data, output files with input data quality check, assembly and annotation results, output SBML models, MEMOTE reports, and BioEMMA map generation results are provided in the project repository: https://github.com/mishakula-group/BioEMMA_article/tree/main/pipeline_three_reconstructors_outputs, accessed on 15 August 2026.

The comparison was performed for three KEGG pathways of central carbon metabolism: glycolysis/gluconeogenesis (map00010), the tricarboxylic acid cycle (map00020), and the pentose phosphate pathway (map00030). For each map, the following parameters were evaluated: the number of reactions in the original KEGG scheme, the number of reactions mapped to the model namespace, the number of reactions retained in each of the three reconstructions, the number of reactions shared by all models, and the number of model-specific reactions. For each KEGG pathway, R_G_, R_M_, and R_R_ denote the sets of mapped KEGG reactions retained after model-based filtering in the gapseq, ModelSEEDpy, and Reconstructor models, respectively. The set of model-specific reactions was defined as follows:

(1) $$\begin{array}{c} R_{specific}=\left[R_{G}\backslash \left(R_{M}\cup {\;R}_{R}\right)\right]\cup \left[R_{M}\backslash \left(R_{G\;}{\cup R}_{R}\right)\right]\cup \left[R_{R}\backslash \left(R_{G}{\cup R}_{M}\right)\right] \end{array}$$

Thus, reactions present in only one of the three reconstructions were considered model-specific. The reaction-by-reaction matrices, union counts, two-model overlaps, and Jaccard values are provided in Supplementary Table S2 and in the project repository: https://github.com/mishakula-group/BioEMMA_article/tree/main/results/table2_reaction_retention, accessed on 15 August 2026.

For additional functional interpretation, FBA fluxes calculated under identical conditions for all three models were overlaid onto the BioEMMA maps. The flux values were used as an additional visualization layer, enabling structural differences between the reconstructions to be compared with the activity of the corresponding reactions under the selected computational scenario. Formal reconstruction quality metrics obtained using MEMOTE were used only as an auxiliary reference point for comparison with the results of the pathway-level analysis. Resulting maps with flux distributions are provided in the project repository: https://github.com/mishakula-group/BioEMMA_article, accessed on 15 August 2026.

#### 2.3.3. Test Case 3: Additional Evaluation Across Diverse Metabolic Models

To evaluate BioEMMA beyond the initial *E. coli* test cases, additional maps were generated for genome-scale metabolic models obtained from the BiGG Models database [17]. The prokaryotic evaluation set included 87 models used for reaction set comparison: 57 *E. coli* reconstructions and 30 models representing other prokaryotic organisms. The manually curated e_coli_core model was generated as an additional reference map but was not included in the pairwise Jaccard summaries. For prokaryotic models, BioEMMA maps were generated for four KEGG pathways: glycolysis/gluconeogenesis (map00010), the citrate cycle (map00020), the pentose phosphate pathway (map00030), and methane metabolism/C1 metabolism (map00680).

For each generated map, retained KEGG reaction identifiers were extracted from the BioEMMA output and compared between models using the Jaccard index calculated over retained reaction sets. For the *E. coli* subset, identical retained reaction sets were additionally grouped to distinguish strain-level reproducibility from genuinely different pathway representations. For the non-*E. coli* prokaryotic subset, pairwise Jaccard similarities, retained reaction counts, and map construction times were analyzed separately. The scripts, model lists, run parameters, generated maps, map statistics, Jaccard tables, and heatmap source files are provided in the project repository: https://github.com/mishakula-group/BioEMMA_article/tree/main/results/prokaryote_maps and https://github.com/mishakula-group/BioEMMA_article/tree/main/results/jaccard, accessed on 15 August 2026.

The methanogenic model *Methanosarcina barkeri i*AF692 was used to test BioEMMA on a specialized pathway, methane metabolism/C1 metabolism (map00680). For this pathway, the number of KEGG reactions, reactions mapped to BiGG identifiers, retained reactions, unresolved reactions, and reactions retained through non-direct matching routes were recorded. The retained reaction set of *i*AF692 was compared with the corresponding sets from the other prokaryotic models using the Jaccard index and reaction frequency analysis. The Jaccard tables, and heatmap source files are provided in the project repository: https://github.com/mishakula-group/BioEMMA_article/tree/main/results/jaccard, accessed on 15 August 2026.

For eukaryotic models, BioEMMA was applied to *Saccharomyces cerevisiae i*MM904, *Mus musculus i*MM1415, and *Homo sapiens* Recon3D using compartment-specific filtering. These models contain 1577, 3726, and 10,600 reactions, respectively. Maps were generated with the BioEMMA compartment parameter, and the number of retained reactions, compartment-specific reaction content, and map construction time were recorded for each model–pathway–compartment combination. The resulting maps are provided in the project repository: https://github.com/mishakula-group/BioEMMA_article/tree/main/results/compartments (accessed on 15 August 2026).

## 3. Results

### 3.1. Automatic Reconstruction of a Pathway Map for a Reference GSM Model

The reference test case comprising the KEGG glycolysis/gluconeogenesis pathway (map00010) and the e_coli_core model, described in Section 2.3, was used to examine the successive transformation of a generic KEGG pathway into a model-specific Escher-compatible map. The analysis focused on identifier mapping, reaction retention after model-based filtering, the incorporation of non-primary metabolites, and visualization of the resulting flux distribution. The original map00010 KGML map contained 56 reactions and 31 metabolites. At the cross-reference mapping stage, 30 reactions (53.6%) and 25 metabolites (80.6%) were mapped to the BiGG namespace. Thus, 26 reactions were not transferred to the subsequent stages because no unambiguous mapping to the model namespace was available. After filtering according to the composition of e_coli_core, 16 of the 30 mapped reactions were retained in the final map, corresponding to 40.0% of the mapped reactions and 21.4% of the original KEGG map. The remaining 18 mapped reactions were excluded because they were absent from the target model. Addition of non-primary stoichiometric metabolites introduced 26 additional nodes.

**Figure 3:**
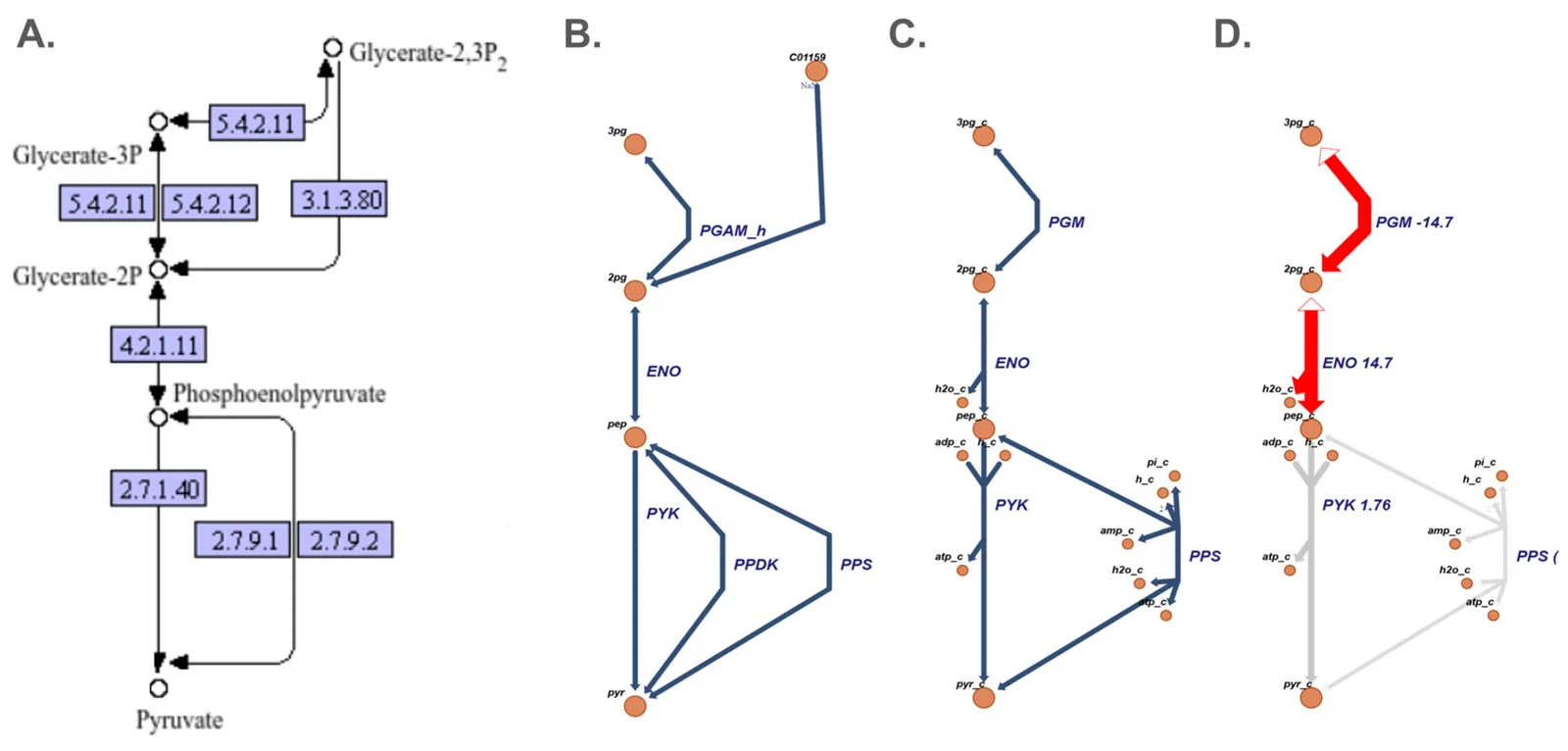
The stages of the automated reconstruction of a glycolysis/gluconeogenesis map00010 fragment for the e_coli_core model using BioEMMA. (**A**) Original fragment of the KEGG Pathway map; (**B**) a map converted to the Escher format while retaining the KGML-derived spatial arrangement; (**C**) a map after filtering reactions according to the model composition and adding non-primary metabolites; (**D**) the final map with overlaid FBA flux values.

Figure 3 shows a fragment of the lower glycolysis pathway (the complete maps are presented in Supplementary Figures S1–S4), which was used to illustrate the main reconstruction stages. In the original KEGG map (Figure 3A), reactions are represented by EC numbers and reflect a generalized scheme of transformations between 3-phosphoglycerate, 2-phosphoglycerate, phosphoenolpyruvate, and pyruvate. After conversion to the Escher format (Figure 3B), the coordinate-based arrangement of the source KEGG pathway is retained, while the reactions are mapped to the corresponding model reactions. At the filtering stage (Figure 3C), transformations absent from e_coli_core are removed from the scheme, including the pyruvate phosphate dikinase reaction (PPDK) and the 2,3-bisphosphoglycerate 3-phosphatase branch. The resulting map retained phosphoglycerate mutase (PGM), enolase (ENO), pyruvate kinase (PYK), and phosphoenolpyruvate synthase (PPS) reactions. Non-primary metabolites required for a stoichiometrically complete representation of the model reactions are additionally added to the map: water, protons, ATP, ADP, AMP, and inorganic phosphate. The final version (Figure 3D) represents a model-specific map with overlaid FBA flux values.

**Figure 4:**
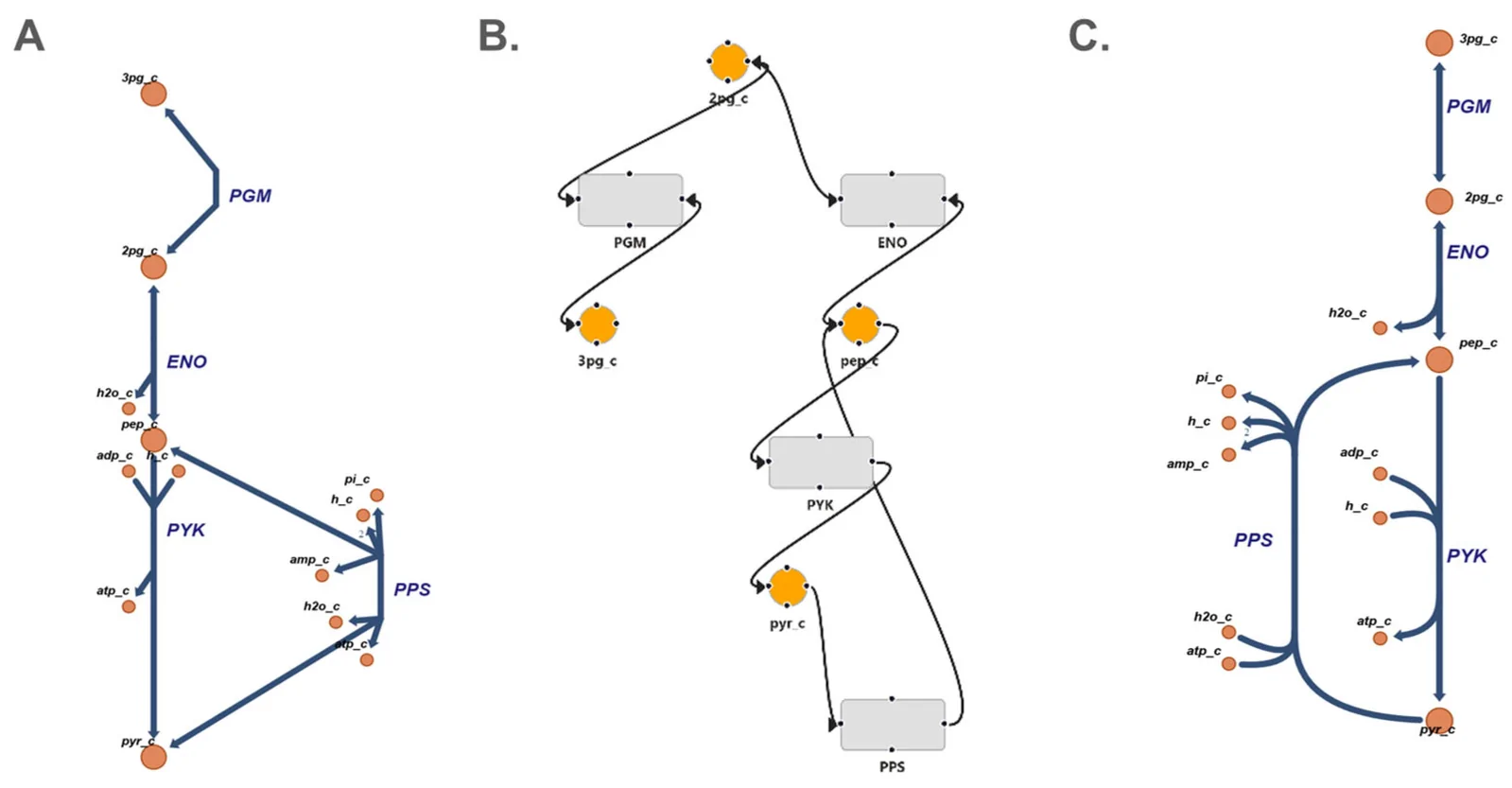
Comparison of glycolysis/gluconeogenesis visualizations (KEGG map00010) for the e_coli_core model. (**A**) Pathway-level map generated by BioEMMA using the KGML-derived coordinate arrangement; (**B**) NAViFluX visualization with an automated topological layout; (**C**) a fragment of the reference e_coli_core central metabolism map manually drawn for Escher [9].

To contextualize the results, BioEMMA was compared with the reference manually curated e_coli_core Escher map, as well as with maps generated by alternative visualization tools using the same input data. The considered solutions can be broadly divided into several groups: manual or predominantly manual maps, which provide high-quality schemes but require expert drawing; semi-manual tools focused on interactive editing and analysis of pre-existing maps; and automated de novo visualization tools, which construct network layouts from the model structure but do not preserve biologically interpretable pathway geometry. BioEMMA was evaluated by projecting the reactions of the e_coli_core model onto the KEGG glycolysis/gluconeogenesis pathway map (map00010), enabling direct comparison with the manually curated Escher reference map and with visualizations generated by alternative tools (Details: Section 2.3). The main comparison is presented in Figure 4: BioEMMA generates a pathway-level map based on the KGML-derived coordinate arrangement (Figure 4A), NAViFLuX [15] produces a visualization with an automated topological layout (Figure 4B), and the manually curated e_coli_core Escher map is used as an expert-prepared representation of the model’s central metabolism (Figure 4C). Visualizations generated using SAMMI, Fluxer, CAVE, Grohar and MetExplore V2 [11,12,13,14,29] are presented in the Supplementary Materials (Supplementary Figures S5–S9).

The comparison demonstrates that the differences between the tools are related not only to the visual appearance of the maps, but also to different formulations of the visualization task. The manually curated Escher map provides the most refined expert representation of central metabolism, but requires prior drawing and cannot be automatically transferred to other models and pathways. Automated de novo approaches, in contrast, allow for rapid visualization directly from the model structure, but generate their own network layouts. Consequently, when models are compared, differences in reaction composition may become confounded with differences in map geometry. BioEMMA instead retains the coordinate arrangement of the source KEGG pathway while filtering and adapting its content to the target model. Consequently, absent reactions, local pathway gaps, and flux-associated differences are displayed at corresponding positions within the same pathway layout.

**Figure 5:**
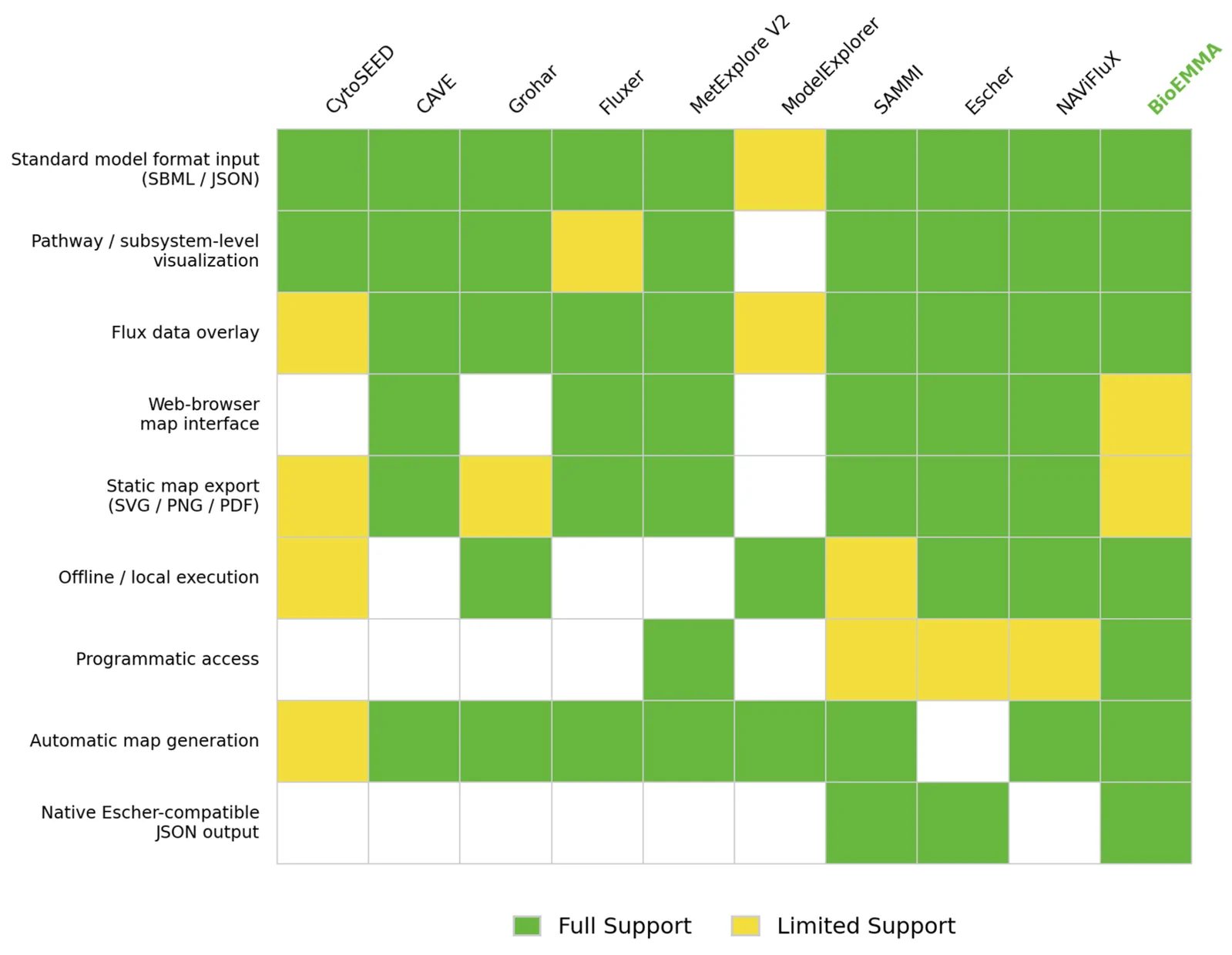
Functional comparison of tools for genome-scale metabolic model visualization and pathway-map generation. Green cells indicate native and documented support, yellow cells indicate limited, indirect, or format-restricted support, and white cells indicate that support was not documented in the evaluated publications or official documentation.

These methodological differences were further summarized in the functional comparison shown in Figure 5. The evaluated criteria included supported model input formats, the level of network visualization, support for flux data overlay, interactivity, export options, availability of a programming interface, and reproducibility of map generation. This comparison was intended to provide functional context rather than a formal ranking because the evaluated tools address overlapping rather than mutually exclusive tasks. Their principal applications can be consolidated into three broad groups: pathway–map generation, layout, or editing (Escher, CAVE, MetExplore V2, Grohar, SAMMI, NAViFluX, and BioEMMA); interactive exploration, analysis, or refinement of metabolic models (CytoSEED, CAVE, MetExplore V2, ModelExplorer, Fluxer, and NAViFluX) and visualization of model-derived flux distributions or overlay of omics data (Escher, CytoSEED, CAVE, MetExplore V2, Grohar, Fluxer, SAMMI, NAViFluX, and BioEMMA). Brief descriptions of the compared tools, definitions of the support categories, and the sources used to assign each category are provided in Supplementary Table S1 and the publication repository: https://github.com/mishakula-group/BioEMMA_article/tree/main/results/fig5_tool_comparison, accessed on 15 August 2026.

The analysis indicates that manual and semi-manual solutions are suitable for preparing high-quality maps and conducting interactive analyses, but require substantial user involvement. Moreover, such approaches are model-specific, and transferring them from one model to another may be difficult, particularly when the models were reconstructed using different tools. Automated layout tools, in turn, are better suited for rapid inspection of model structure, but often do not provide a common spatial framework when several reconstructions are compared. Some solutions are designed for visualization of the entire metabolic network or large subnetworks, which is useful for global inspection but less convenient for detailed pathway-level comparison of specific metabolic pathways.

Thus, BioEMMA addresses a narrower but critically important use case that is only partially covered by the tools considered: it combines automated map generation, pathway-level representation, the use of pathway-level representation based on curated KEGG pathway layouts, compatibility with flux data, and export to the Escher JSON and HTML format. This combination is important for reproducible model comparison because metabolic maps of different reconstructions are generated within the same coordinate system. Therefore, the common pathway layout enables direct comparison of local differences between models, such as missing reactions, model-specific pathway regions, and differences in predicted flux distributions.

### 3.2. Pathway-Level Comparison of Alternative E. coli GSM Reconstructions

Following the verification of BioEMMA using a single reference model, the next test case was aimed at evaluating the main analytical capability of the tool: comparison of multiple reconstructions within a common spatial coordinate system. Traditionally, alternative GSM reconstructions can be compared using global quality metrics, including MEMOTE reports [8], which enable assessment of model consistency, annotation completeness, the presence of technical errors, and comparative quality summaries across different reconstructions. However, such metrics do not indicate which specific reactions, branches, or pathway regions differ between models or how these differences may affect flux distributions.

Scenario III was applied to the *E. coli* raw data described in Section 2.3, generating three alternative reconstructions using gapseq, ModelSEEDpy, and Reconstructor [3,6,7]. This design allowed differences between the reconstruction tools to be considered not only as differences in reaction counts or aggregate quality metrics, but also as local events within specific pathways: retention or absence of a reaction, the appearance of a model-specific element, or differences in reaction activity under FBA.

For each model, BioEMMA maps were generated for three KEGG pathways of central carbon metabolism: glycolysis/gluconeogenesis (map00010), the tricarboxylic acid cycle (map00020), and the pentose phosphate pathway (map00030).

At the stage of mapping the original KEGG maps to the model namespace, 47 of 56 reactions were identified for map00010, 21 of 29 reactions for map00020, and 55 of 59 reactions for map00030 (Table 2). Thus, for all three pathways, most reactions in the original scheme could be mapped to model reactions. However, after filtering according to the composition of the individual reconstructions, only a subset of these reactions was retained, and the degree of retention differed markedly between the tools (Table 2).

Normalization of pathway coverage to the number of mapped reactions made it possible to separate the contribution of cross-reference mapping from the differences between the reconstruction tools themselves. After excluding reactions that could not be mapped to the model namespace, gapseq retained the largest proportion of reactions in all three pathways: 59.6% of mapped reactions for map00010, 81.0% for map00020, and 63.6% for map00030. ModelSEEDpy, in contrast, generated the most compact reconstructions of the central pathways, retaining 38.3%, 52.4%, and 41.8% of mapped reactions, respectively. Reconstructor occupied an intermediate position for map00020 and map00030, but showed the same coverage as gapseq for map00010. Thus, BioEMMA revealed not only an isolated discrepancy between the models, but pathway-specific differences: gapseq included a broader set of central metabolic reactions, whereas ModelSEEDpy included a more limited set. The tricarboxylic acid cycle was particularly informative. For map00020, 21 KEGG reactions were mapped, of which nine were model-specific, corresponding to 42.9% of the mapped reaction space. This indicates that, in this case, differences between the reconstruction tools affected not only the completeness of pathway coverage but also the composition of alternative reactions within a central energy metabolism pathway.

The same reaction sets were also compared using pairwise Jaccard similarity. For map00010, the Jaccard index was 0.500 between gapseq and ModelSEEDpy, 0.657 between gapseq and Reconstructor, and 0.643 between ModelSEEDpy and Reconstructor. For map00020, the corresponding values were 0.733, 0.611, and 0.786, whereas for map00030 they were 0.622, 0.575, and 0.690 (Supplementary Table S2). These values indicate that the three reconstructions retained a shared central metabolism core, but the overlap was only partial and depended on the pathway and reconstruction tool. The strongest divergence was observed for the gapseq-ModelSEEDpy comparison in glycolysis/gluconeogenesis and for the gapseq-Reconstructor comparison in the pentose phosphate pathway, whereas the TCA cycle showed higher overall similarity which corresponds to model-specific reactions in Table 2.

Comparison with the MEMOTE report (Supplementary Figure S10) showed that the quality assessment was generally consistent with the BioEMMA results. The gapseq and Reconstructor models had similarly high-quality scores (81% and 84% of the “total score”, respectively), whereas the ModelSEEDpy model was characterized by a substantially lower “total score”—41%. At the same time, the overall size of the reconstructions did not directly correspond to their coverage of specific pathways. The gapseq model contained the largest number of reactions (3011) and also showed the highest coverage of all three analyzed KEGG maps. However, ModelSEEDpy contained more reactions than Reconstructor (1779 and 1527, respectively), while retaining a smaller proportion of mapped reactions in each of the three central pathways. This difference is particularly important for the interpretation of automated reconstruction results. Based solely on general model statistics, one might expect a larger reconstruction to cover more reactions within individual metabolic pathways. The BioEMMA results demonstrate that this assumption is only partially valid. Thus, BioEMMA adds not only visualization to MEMOTE but also a distinct analytical layer: neither a high score nor a larger number of reactions alone indicates which central pathways are covered more or less completely, whereas BioEMMA localizes these differences.

On the other hand, structural pathway coverage does not indicate that the retained reactions are used under a particular computational condition. Therefore, the next step was the automated overlay of FBA fluxes onto the BioEMMA maps. This layer made it possible to distinguish three reaction states within the same pathway scheme: the absence of the reaction from the model, the presence of the reaction with zero flux, and the presence of the reaction with nonzero flux. Map00020 was selected for this analysis because this pathway exhibited the highest proportion of model-specific reactions.

**Figure 6:**
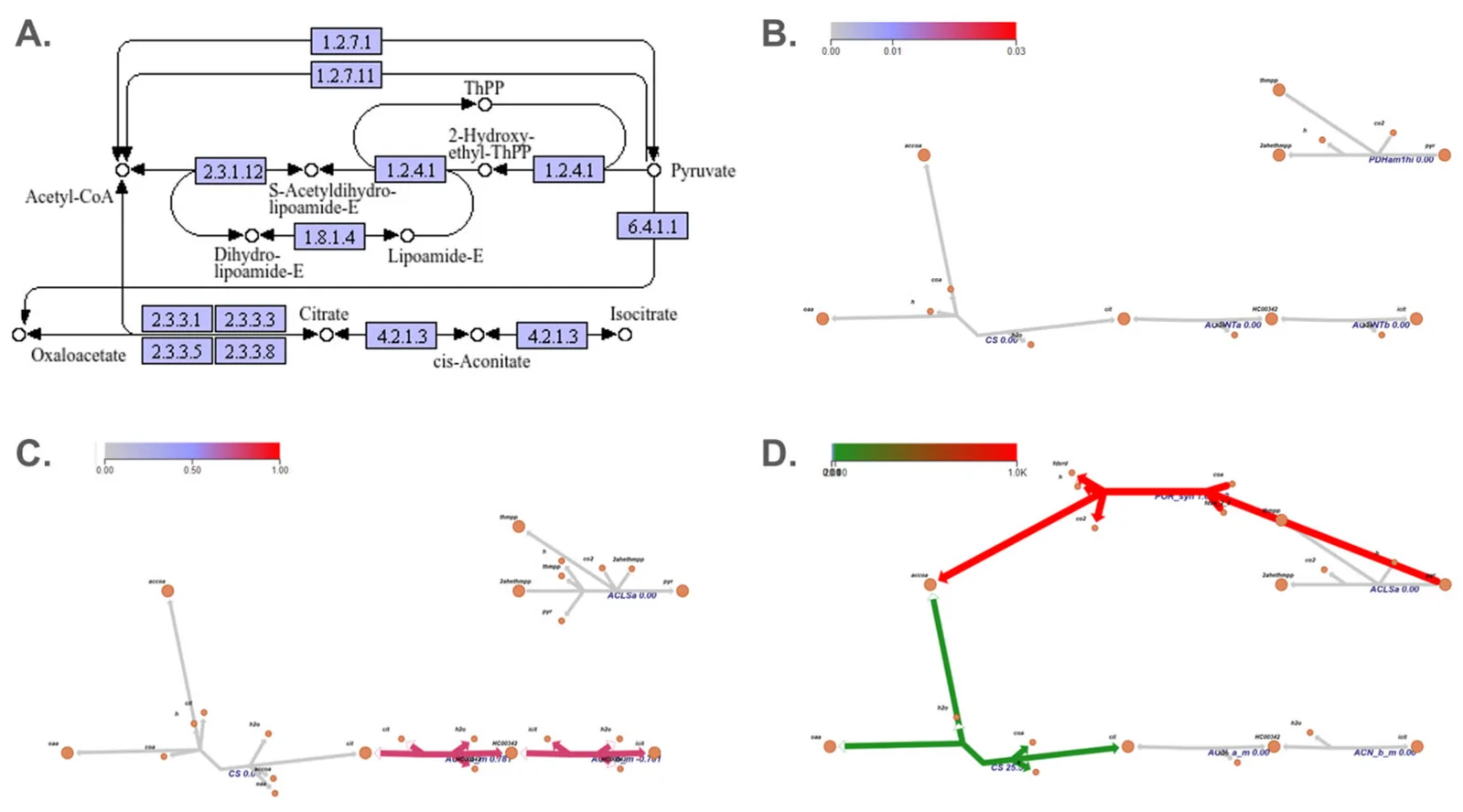
A fragment of the reconstructed metabolic pathway map00020 with overlaid calculated fluxes for the three models. (**A**) The original KEGG scheme; (**B**–**D**) the corresponding BioEMMA maps for the gapseq, ModelSEEDpy, and Reconstructor models, respectively. Edge color and thickness reflect the magnitude of the FBA flux, allowing for comparison of reaction activity within the fragment across different reconstructions. The heatmap was added by Escher automatically, based on the total values of fluxes on displayed region for each model.

Figure 6 presents a part of the TCA map (map00020). In the original KEGG map this region includes reactions that connect pyruvate, acetyl-CoA, citrate, cis-aconitate and iso-citrate. The full reconstructed maps are available in Supplementary Materials Figures S11–S14 or by the following link (https://github.com/mishakula-group/BioEMMA_article/tree/main/results/figure_03/map00020, accessed on 15 August 2026). The three *E. coli* reconstructions were analyzed, taking into account that reconstruction and gap filling were performed on the M9_vit_aa medium, from the gapseq medium database, which contains glucose as the main carbon source, ammonium, inorganic salts, trace ions, vitamins, and amino acids and anaerobic growth conditions.

Experimental ^13^C metabolic flux analysis has shown that *E. coli* central metabolism differs substantially between aerobic and anaerobic glucose growth: under aerobic conditions the oxidative TCA cycle is active, whereas under anaerobic glucose conditions the TCA cycle becomes bifurcated, with separated oxidative and reductive branches rather than a fully cyclic respiratory mode [30]. Analysis of the generated BioEMMA maps shows that the three reconstructions showed activity of only part of the TCA, but the flux patterns between them differed. Gapseq showed activity in selected PEP/OAA- and succinate-associated reactions, but did not show a pronounced flux through citrate synthase and the citrate–isocitrate segment (Supplementary Figure S12). Reconstructor showed stronger activity around citrate formation and selected malate/OAA/PEP-associated reactions, whereas ModelSEEDpy showed activity around the citrate/isocitrate/aconitate region despite inactive citrate synthase (Figure 6). As a result, the maps demonstrate that BioEMMA can reveal reconstruction-specific differences in the local flux distribution of the TCA and can help identify patterns that require further inspection of exchange bounds, reaction directionality, and possible internal cycles.

### 3.3. Evaluation of BioEMMA Across Diverse Metabolic Models

BioEMMA was further evaluated on models differing in organism identity, pathway specialization, model size, and compartmental structure. This comparison was designed to test whether the same KEGG-based map generation strategy remains reproducible for closely related models, sensitive to biological and reconstruction-specific differences between organisms, applicable to specialized metabolic pathways, and scalable to eukaryotic models, which can have the same metabolic pathway indifferent compartments.

#### 3.3.1. Application to Diverse Prokaryotic Models

Across the four evaluated prokaryotic pathways, *E. coli* reconstructions showed highly reproducible retained reaction sets. The median pairwise Jaccard index was 1.000 for glycolysis/gluconeogenesis, 1.000 for the citrate cycle, 0.955 for the pentose phosphate pathway, and 1.000 for methane metabolism/C1 metabolism. Median retained reaction counts were 15, 7, 21, and 21 reactions, respectively. In contrast, the 30 non-*E. coli* prokaryotic models showed broader between-model variation, with median Jaccard indices of 0.636, 0.571, 0.650, and 0.647 for the same four pathways (Supplementary Table S3). Median retained reaction counts in this subset were 14, 7, 19.5, and 17 reactions, respectively. Thus, this analysis showed both reproducibility for closely related models and sensitivity to organism- and reconstruction-specific pathway content.

Glycolysis/gluconeogenesis (map00010) was then analyzed in more detail as a representative central carbon metabolism pathway. Among 57 *E. coli* reconstructions, BioEMMA generated highly similar maps: the median pairwise Jaccard index was 1.000, the mean Jaccard index was 0.973, and 63.4% of model pairs had identical retained reaction sets. The retained maps contained 13–15 reactions, with 12 reactions shared by all *E. coli* models and three variable KEGG reactions across the subset. The 44 models retained all mapped 15 reactions (Supplementary Table S3). A second group of 12 models retained 14 reactions and differed mainly by the absence of R00710, the NAD+-dependent oxidation of acetaldehyde to acetate. The remaining model, *i*JR904, retained 13 reactions and lacked R00711 and R00754 which corresponding to NADP+-dependent and NAD+-dependent ethanol–acetaldehyde interconversion reaction (Supplementary Table S3). Thus, differences between *E. coli* maps were restricted to a small number of reactions and associated with more lumped representation of ethanol metabolism which is associated with models specify.

The same pathway was then analyzed across 30 non-*E. coli* prokaryotic models. In contrast to the *E. coli* subset, these models showed broader variation: the median pairwise Jaccard index was 0.636, with values ranging from 0.364 to 1.000 (Supplementary Table S3). Retained reaction counts ranged from 10 to 22 reactions, with a median of 14 reactions. Therefore, pathway coverage remained sufficient for comparison, while the retained reaction sets reflected organism- and reconstruction-specific differences.

#### 3.3.2. Visualization of a Specialized Metabolic Pathway

BioEMMA was also tested on methane metabolism/C1 metabolism (map00680), using the methanogenic archaeal model *Methanosarcina barkeri i*AF692 as the primary example. This pathway contains broadly distributed C1 metabolism reactions as well as reactions characteristic of methanogenic metabolism. For *i*AF692, 31 of 104 KEGG reactions on map00680 were retained in the generated model-specific map, corresponding to 29.8% reaction retention. The map also retained 57 of 86 KEGG metabolites, corresponding to 66.3% metabolite retention.

Comparison of *i*AF692 with the other prokaryotic models showed that the retained map00680 reaction set was not simply a generic C1 metabolism map. Pairwise Jaccard similarity between *i*AF692 and the other 86 prokaryotic models had a median value of 0.300 and ranged from 0.265 to 0.514 (Supplementary Table S3). Eleven reactions retained in *i*AF692 were not retained in any other model in the evaluation set: R03464, R05774, R08217, R09098, R09124, R09998, R09999, R10392, R10393, R10395, and R10396. At the same time, partial overlap with other models was retained through formaldehyde and general C1 metabolism reactions. Thus, BioEMMA can visualize both the shared C1 metabolism component of map00680 and the methanogen-specific pathway content represented in *i*AF692.

#### 3.3.3. Application for Visualization of Eukaryotic Models

Finally, BioEMMA was applied to eukaryotic models of increasing size: *i*MM904, *i*MM1415, and Recon3D, containing 1577, 3726, and 10,600 reactions, respectively. The main feature of these models is the presence of the same metabolic pathways in the different compartments. For the same four pathways used in the prokaryotic analysis, all 12 model–pathway maps were generated successfully. Mean map construction time increased with model size, from 3.05 s for *i*MM904 to 4.08 s for *i*MM1415 and 8.13 s for Recon3D (further details in Section 2.3.3). Across the three eukaryotic models, retained reaction counts increased with model size for all four pathways. For *i*MM904, BioEMMA retained 15, 7, 11, and 14 reactions for map00010, map00020, map00030, and map00680, respectively. For *i*MM1415, the corresponding counts were 16, 9, 13, and 15 reactions, and for Recon3D they were 17, 13, 15, and 19 reactions. Pairwise Jaccard analysis showed moderate to high similarity between the three eukaryotic models for glycolysis/gluconeogenesis, the pentose phosphate pathway, and methane metabolism/C1 metabolism, with mean Jaccard indices of 0.814, 0.815, and 0.780, respectively. The citrate cycle showed stronger model-specific variation, with a mean Jaccard index of 0.574.

Together, these analyses show that BioEMMA generates reproducible maps for closely related models, preserves biologically meaningful differences between diverse prokaryotes, captures specialized pathway content in a methanogenic model, and scales to eukaryotic reconstructions with thousands of reactions and different compartments.

## 4. Discussion

The results of this study highlight a central trade-off in automated pathway–map generation for genome-scale metabolic models. Fully manual pathway maps, such as those created in Escher, can achieve high visual quality and biological clarity, but they do not scale well when multiple organisms, reconstruction tools, or pathway variants need to be compared. Fully automatic graph layouts, in contrast, are scalable but often generate model-specific geometries that make visual comparison between alternative reconstructions difficult. BioEMMA addresses this gap by using KEGG pathway maps as a fixed spatial scaffold and converting them into model-specific Escher-compatible maps. This strategy helps retain a recognizable pathway arrangement while allowing model content and flux values to be represented in an editable GSM-oriented format. The comparison of alternative reconstructions illustrates why such pathway-level projections are useful. Recent benchmarking studies have shown that automated reconstruction tools differ in database content, annotation transfer, GPR assignment, transporter prediction, gap-filling strategies, and phenotype prediction accuracy [6,31]. These differences are not necessarily distributed uniformly across the whole model. A larger reaction set may indicate broader network scope, but it does not guarantee better representation of a particular metabolic subsystem. This is consistent with another recent work emphasizing that large GSMs can be difficult to analyze and visualize, whereas compact representations of central energy and biosynthetic metabolism may provide higher interpretability for defined biological questions [32]. BioEMMA therefore complements global model statistics by showing where differences between reconstructions occur within a specific pathway.

This also defines the relationship between BioEMMA and global quality-control frameworks. MEMOTE provides standardized tests for formal GSM properties, including annotation coverage, stoichiometric consistency, mass and charge balance, and related technical features [8]. These tests are essential for evaluating model quality, but they assess the reconstruction primarily as a whole. BioEMMA adds a local interpretive layer by showing which reactions are retained, missing, or reconstruction-specific in a defined biochemical context. The FBA overlay functionality extends this comparison further: it distinguishes reactions that are merely present in the model from reactions that carry flux under a given set of constraints. This is relevant to broader discussions of model validation and model selection in constraint-based modeling, where alternative network structures should be evaluated not only by formal consistency but also by their consequences for predicted flux states [33].

The broader model list from BiGG database further showed that this pathway-level interpretive framework is not restricted to alternative *E. coli* reconstructions or to central carbon metabolism. The high agreement among *E. coli* models and the broader, yet biologically interpretable, variation observed across non-*E. coli* prokaryotes models indicate that BioEMMA preserves model-specific reaction content while projecting different reconstructions onto a common KEGG-derived spatial framework. This capability was also shown for methane metabolism, where the *i*AF692 reconstruction retained both broadly shared C1 metabolism reactions and methanogen-associated reactions absent from the other evaluated models. The examples on the eukaryotic models further extend this approach to metabolic networks containing multiple subcellular compartments. Compartment-specific filtering allows cytosolic, mitochondrial, and other compartment-specific reaction variants to be examined separately while retaining a common pathway layout. The observed construction times for maps ranging from the yeast model *i*MM904 to the substantially larger human Recon3D model indicate that the approach remains computationally tractable for most part of existing eukaryotic models. However, the reliability of these cross-organism and cross-compartment projections ultimately depends on the quality and completeness of reaction identifier reconciliation.

The main limitation of the approach is identifier reconciliation between biochemical databases. Automatic mapping between KEGG, MetaNetX, BiGG, SEED, and other namespaces is version-dependent and can be affected by differences in reaction granularity, database curation rules, and intermediate identifiers. MetaNetX/MNXref was developed to reconcile metabolites and biochemical reactions across major biochemical and GSM-related resources, but it cannot remove all ambiguity between independently curated databases [34]. The example of this limitation we find on map00010. We use glycolysis for test case because it is one of the most extensively studied and consistently annotated metabolic pathways. Nevertheless, during manual inspection of the generated BioEMMA map, the glyceraldehyde-3-phosphate dehydrogenase reaction was identified as an expected but unmapped element. This reaction is present in e_coli_core as the BiGG reaction GAPD and is annotated with KEGG (R01061) and MetaNetX (MNXR100040). However, in the cross-reference table used for this BioEMMA run, R01061 was associated with another MetaNetX reaction identifier that did not provide a BiGG correspondence. Therefore, the absence of the GAPD label on the map reflected the specific state of the KEGG–MetaNetX–BiGG cross-reference layer rather than the absence of the reaction from the model. This case demonstrates that even for well-characterized central metabolic pathways, unresolved or version-dependent identifier mappings can affect automatically generated maps. Another example is related to the methane metabolism map. The *i*AF692 model contains methyl-coenzyme M reductase as the model reaction MCR, but this reaction was not retained on the generated map because the corresponding KEGG-map reaction is linked to a different database record. This case also demonstrates that even for biologically well-defined reactions, database-level differences in identifiers and reaction granularity can affect automatically generated maps. Taken together, these examples show that the absence of a reaction from a generated BioEMMA map cannot be interpreted directly as its biological absence from the source model. Map retention depends not only on model content but also on the completeness and concordance of the cross-reference chains linking KEGG and MetaNetX to BiGG or ModelSEED. Researchers should therefore take this limitation into account when analyzing and interpreting generated maps. To understand the total coverage of ModelSEED and BiGG IDs to KEGG maps we made the pathway-wide mapping analysis which shows that this limitation is pathway-dependent rather than uniform. After excluding KEGG maps without reaction entries, most reaction-containing pathways had usable cross-reference coverage: ~74.5% of KEGG reactions across 153 pathways could be mapped to either ModelSEED or BiGG (https://github.com/mishakula-group/BioEMMA_article/blob/main/results/table_02_kegg_database_mapping/kegg_database_mapping_all_pathways.md, accessed on 15 August 2026). Coverage was particularly strong for broadly conserved metabolic subsystems, including carbohydrate, amino acid, nucleotide, and central carbon metabolism, supporting the use of BioEMMA for core metabolic analyses. However, coverage was weaker for BiGG-only mappings and for several specialized pathway classes, especially glycan-related pathways. Therefore, specialized or poorly cross-referenced pathways may require additional mapping curation or user-defined correction tables before final map generation.

Another limitation is local layout quality. KGML coordinates do not always correspond to ideal reaction centers in an Escher-style graph. They may reflect enzyme labels, EC numbers, or other graphical elements in the original KEGG diagram. In addition, non-primary stoichiometric metabolites such as water, protons, phosphate, ATP, ADP, AMP, and cofactors are often absent from KEGG diagrams and must be placed heuristically. These factors can produce shifted connectors, dense local regions, or suboptimal segment geometry even when the global pathway organization remains recognizable. The use of Escher map as an output partially mitigates this limitation, because the automatically generated map remains editable: users can correct labels, adjust local geometry, simplify dense regions, and refine the layout to publication quality.

A further practical limitation is that BioEMMA does not currently perform FVA, gene essentiality analysis, or multi-omics preprocessing and visualization. Nevertheless, results of these performed analyses can be visualized using the generated Escher HTML maps when provided in a compatible format. BioEMMA also does not currently provide a standalone graphical interface. Its implementation instead emphasizes reproducible command-line execution, facilitating integration into automated model reconstruction and analysis pipelines. At the same time, its use through the BioUML web interface enables workflow execution without command–line interaction, whereas the resulting Escher HTML maps provide an interactive environment for map exploration, editing, and data overlay. Thus, the current implementation offers complementary programmatic and web-based access routes, although more direct integration with external analyses and simplified data import procedures remains directions for future development.

## 5. Conclusions

In this study, BioEMMA was developed as a tool for the automated generation of model-specific metabolic pathway maps in the Escher JSON format using KGML-derived coordinate arrangement. The results demonstrate that representation within a common KGML-derived coordinate framework provides a practical basis for pathway-level comparison of alternative genome-scale metabolic reconstructions. In the analyzed models, this representation localized differences in reaction coverage and distinguished reactions absent from a model from those present but inactive under the selected constraints. It therefore provides information complementary to model statistics and formal quality metrics and can support targeted inspection of pathway regions during model assessment and curation.

The reliability of such comparisons depends primarily on the completeness and consistency of cross-references between biochemical databases and on the quality of automatically generated local layouts. For pathways with sufficient identifier coverage, BioEMMA provides reproducible and editable model-specific maps that can be incorporated into automated reconstruction workflows. For pathways with lower cross-reference coverage, BioEMMA can give a basic template, which can be further manually curated and modified, for more accurate interpretation.

## Figures and Tables

**Table 1:** List of tools used for pipeline development.

Pipeline Stage	Tool	Version	Reference
Data preprocessing	fastp	0.24.0	[21]
Raw and trimmed reads quality	FastQC	0.11.9	[22]
Bacterial genome assembly	SPAdes	4.1.0	[23]
Quality assessment of the assembled genome	QUAST	5.3	[24]
Bacterial genome annotation	Prokka	1.15.6	[25]
Automated model reconstruction	Bactabolize	1.0.3	[26]
	Reconstructor	1.1.1	[7]
	ModelSEEDpy	0.4.2	[3]
	gapseq	2.1.0	[6]
Quality assessment of the reconstructed model	MEMOTE	0.11.1	[8]
Visualization	BioEMMA	0.4.3	Current work

**Table 2:** Completeness of central metabolic pathway reconstruction relative to the original KEGG maps.

KEGG Pathway	Map00010	Map00020	Map00030
Reactions in the KEGG map	56	29	59
Mapped reactions	47 (83.9%)	21 (72.4%)	55 (93.2%)
Retained in gapseq, *n* (% of mapped reactions)	28 (59.6%)	17 (81.0%)	35 (63.6%)
Retained in ModelSEEDpy, *n* (% of mapped reactions)	18 (38.3%)	11 (52.4%)	23 (41.8%)
Retained in Reconstructor, *n* (% of mapped reactions)	28 (59.6%)	14 (66.6%)	26 (47.3%)
Reactions shared by all three models	16	11	20
Model-specific reactions ^1^	12	9	14

^1^ Model-specific reactions were defined as mapped KEGG reactions retained after model-based filtering in exactly one of the three reconstructions, as formally specified in Equation (1) in Section 2.3.2.

## Data Availability

The developed BioEMMA tool is available at https://github.com/KAVladD/BioEMMA (accessed on 21 August 2026). The results presented in this article, including the source models, outputs of the reconstruction pipeline, and maps generated using BioEMMA, are available in the GitHub repository at https://github.com/mishakula-group/BioEMMA_article (accessed on 21 August 2026).

## Supplementary Materials

The following supporting information can be downloaded at https://www.mdpi.com/article/10.3390/biology15171493/s1, Figure S1: KEGG reference map for glycolysis/gluconeogenesis (map00010); Figure S2: Escher visualization of the KEGG-derived glycolysis/gluconeogenesis map (map00010); Figure S3: BioEMMA model-specific glycolysis/gluconeogenesis map (map00010); Figure S4: BioEMMA model-specific glycolysis/gluconeogenesis map with flux overlay (map00010); Figure S5: SAMMI visualization of the *E. coli* core model; Figure S6: Fluxer visualization of the *E. coli* core model, provided as PNG and SVG files; Figure S7: CAVE visualization of the D-glucose pathway, provided as JPG and SVG files; Figure S8: Grohar visualization of the *E. coli* core model; Figure S9: MetExplore V2 visualization of the *E. coli* core model; Figure S10: MEMOTE comparison report between gapseq, ModelSEEDpy and Reconstructor GSM reconstructions; Figure S11: KEGG reference map for the citrate cycle (map00020); Figure S12: BioEMMA map00020 visualization for the gapseq reconstruction; Figure S13: BioEMMA map00020 visualization for the ModelSEEDpy reconstruction; Figure S14: BioEMMA map00020 visualization for the Reconstructor output; Table S1: Tool descriptions and criteria used for the functional comparison of metabolic model visualization tools; Table S2: Reaction presence/absence matrices, union counts, two-model overlaps, and pairwise Jaccard similarities for the pathways compared in Table 2; Table S3: Model lists, retained reaction counts, Jaccard summaries, compartment-specific maps, flux tables, and map construction times for the diverse-model evaluation.

## Abbreviations

The following abbreviations are used in this manuscript:

ADP	adenosine diphosphate
AMP	adenosine monophosphate
ATP	adenosine triphosphate
CoA	coenzyme A
FBA	flux balance analysis
GAPD	glyceraldehyde-3-phosphate dehydrogenase
GSM	genome-scale metabolic model
GPR	gene–protein–reaction
MCR	methyl-coenzyme M reductase
OAA	oxaloacetate
PEP	phosphoenolpyruvate
TCA	tricarboxylic acid cycle

## References

[1] Orth J.D., Thiele I., Palsson B.Ø. (2010). What Is Flux Balance Analysis?. Nat. Biotechnol..

[2] Thiele I., Palsson B.Ø. (2010). A Protocol for Generating a High-Quality Genome-Scale Metabolic Reconstruction. Nat. Protoc..

[3] Seaver S.M.D., Liu F., Zhang Q., Jeffryes J., Faria J.P., Edirisinghe J.N., Mundy M., Chia N., Noor E., Beber M.E. (2021). The ModelSEED Biochemistry Database for the Integration of Metabolic Annotations and the Reconstruction, Comparison and Analysis of Metabolic Models for Plants, Fungi and Microbes. Nucleic Acids Res..

[4] Wood-Charlson E.M., Henry C.S., Dehal P.S., Mahmud G., Allen B.H., Beilsmith K., Blair D.D., Canon S., Cashman M., Chivian D. (2026). KBase: Open-Source Platform for Collaborative Biological Data Analysis and Publication. J. Mol. Biol..

[5] Machado D., Andrejev S., Tramontano M., Patil K.R. (2018). Fast Automated Reconstruction of Genome-Scale Metabolic Models for Microbial Species and Communities. Nucleic Acids Res..

[6] Zimmermann J., Kaleta C., Waschina S. (2021). Gapseq: Informed Prediction of Bacterial Metabolic Pathways and Reconstruction of Accurate Metabolic Models. Genome Biol..

[7] Jenior M.L., Glass E.M., Papin J.A. (2023). Reconstructor: A COBRApy Compatible Tool for Automated Genome-Scale Metabolic Network Reconstruction with Parsimonious Flux-Based Gap-Filling. Bioinformatics.

[8] Lieven C., Beber M.E., Olivier B.G., Bergmann F.T., Ataman M., Babaei P., Bartell J.A., Blank L.M., Chauhan S., Correia K. (2020). MEMOTE for Standardized Genome-Scale Metabolic Model Testing. Nat. Biotechnol..

[9] King Z.A., Dräger A., Ebrahim A., Sonnenschein N., Lewis N.E., Palsson B.O. (2015). Escher: A Web Application for Building, Sharing, and Embedding Data-Rich Visualizations of Biological Pathways. PLoS Comput. Biol..

[10] Rowe E., Palsson B.O., King Z.A. (2018). Escher-FBA: A Web Application for Interactive Flux Balance Analysis. BMC Syst. Biol..

[11] Schultz A., Akbani R. (2020). SAMMI: A Semi-Automated Tool for the Visualization of Metabolic Networks. Bioinformatics.

[12] Hari A., Lobo D. (2020). Fluxer: A Web Application to Compute, Analyze and Visualize Genome-Scale Metabolic Flux Networks. Nucleic Acids Res..

[13] Cottret L., Frainay C., Chazalviel M., Cabanettes F., Gloaguen Y., Camenen E., Merlet B., Heux S., Portais J.-C., Poupin N. (2018). MetExplore: Collaborative Edition and Exploration of Metabolic Networks. Nucleic Acids Res..

[14] Mao Z., Yuan Q., Li H., Zhang Y., Huang Y., Yang C., Wang R., Yang Y., Wu Y., Yang S. (2023). CAVE: A Cloud-Based Platform for Analysis and Visualization of Metabolic Pathways. Nucleic Acids Res..

[15] Krishnamurthy M.B., Harish P.S., Subramanian A. (2026). NAViFluX: A Visualization-centric Platform for Interactive Analysis, Refinement and Design of Genome-scale Metabolic Networks. Bioinformatics.

[16] Kanehisa M., Furumichi M., Sato Y., Matsuura Y., Ishiguro-Watanabe M. (2025). KEGG: Biological Systems Database as a Model of the Real World. Nucleic Acids Res..

[17] Norsigian C.J., Pusarla N., McConn J.L., Yurkovich J.T., Dräger A., Palsson B.O., King Z. (2020). BiGG Models 2020: Multi-Strain Genome-Scale Models and Expansion across the Phylogenetic Tree. Nucleic Acids Res..

[18] Moretti S., Niknejad A., Pagni M., Mehl F. (2026). MetaNetX: A Bridge between Metabolic Resources for Enhanced Curation and Multi-Omics Data Harmonization. Nucleic Acids Res..

[19] Ebrahim A., Lerman J.A., Palsson B.O., Hyduke D.R. (2013). COBRApy: COnstraints-Based Reconstruction and Analysis for Python. BMC Syst. Biol..

[20] Kolpakov F., Akberdin I., Kiselev I., Kolmykov S., Kondrakhin Y., Kulyashov M., Kutumova E., Pintus S., Ryabova A., Sharipov R. (2022). BioUML—Towards a Universal Research Platform. Nucleic Acids Res..

[21] Chen S., Zhou Y., Chen Y., Gu J. (2018). Fastp: An Ultra-Fast All-in-One FASTQ Preprocessor. Bioinformatics.

[22] Andrews S. (2010). FastQC: A Quality Control Tool for High Throughput Sequence Data. Babraham Bioinformatics. https://www.bioinformatics.babraham.ac.uk/projects/fastqc/.

[23] Prjibelski A., Antipov D., Meleshko D., Lapidus A., Korobeynikov A. (2020). Using SPAdes De Novo Assembler. Curr. Protoc. Bioinform..

[24] Gurevich A., Saveliev V., Vyahhi N., Tesler G. (2013). QUAST: Quality Assessment Tool for Genome Assemblies. Bioinformatics.

[25] Seemann T. (2014). Prokka: Rapid Prokaryotic Genome Annotation. Bioinformatics.

[26] Vezina B., Watts S.C., Hawkey J., Cooper H.B., Judd L.M., Jenney A.W., Monk J.M., Holt K.E., Wyres K.L. (2023). Bactabolize Is a Tool for High-Throughput Generation of Bacterial Strain-Specific Metabolic Models. eLife.

[27] DeJongh M., Bockstege B., Frybarger P., Hazekamp N., Kammeraad J., McGeehan T. (2012). CytoSEED: A Cytoscape Plugin for Viewing, Manipulating and Analyzing Metabolic Models Created by the Model SEED. Bioinformatics.

[28] Martyushenko N., Almaas E. (2019). ModelExplorer—Software for Visual Inspection and Inconsistency Correction of Genome-Scale Metabolic Reconstructions. BMC Bioinform..

[29] Moškon M., Zimic N., Mraz M. (2018). Grohar: Automated Visualization of Genome-Scale Metabolic Models and Their Pathways. J. Comput. Biol..

[30] Gonzalez J.E., Long C.P., Antoniewicz M.R. (2017). Comprehensive Analysis of Glucose and Xylose Metabolism in Escherichia Coli under Aerobic and Anaerobic Conditions by 13C Metabolic Flux Analysis. Metab. Eng..

[31] Mendoza S.N., Olivier B.G., Molenaar D., Teusink B. (2019). A Systematic Assessment of Current Genome-Scale Metabolic Reconstruction Tools. Genome Biol..

[32] Corrao M., He H., Liebermeister W., Noor E., Bar-Even A. (2025). A Compact Model of Escherichia Coli Core and Biosynthetic Metabolism. PLoS Comput. Biol..

[33] Kaste J.A.M., Shachar-Hill Y. (2024). Model Validation and Selection in Metabolic Flux Analysis and Flux Balance Analysis. Biotechnol. Prog..

[34] Moretti S., Tran V.D.T., Mehl F., Ibberson M., Pagni M. (2021). MetaNetX/MNXref: Unified Namespace for Metabolites and Biochemical Reactions in the Context of Metabolic Models. Nucleic Acids Res..

